# Supplementary material for: Transcriptomic Insights into Mechanisms of Early Seed Maturation in the Garden Pea (Pisum sativum L.)
Source: Cells. 2020 Mar 23;9(3):779. doi: 10.3390/cells9030779 (PMC7140803; doi:10.3390/cells9030779)
Supplement: Supplementary file 1 [file cells-09-00779-s001.zip › Supplementary materials/FileS2.zip!/FileS1/Supporting Maps&Profiles/Topology Profiles.pdf]

Number of activated spot modules

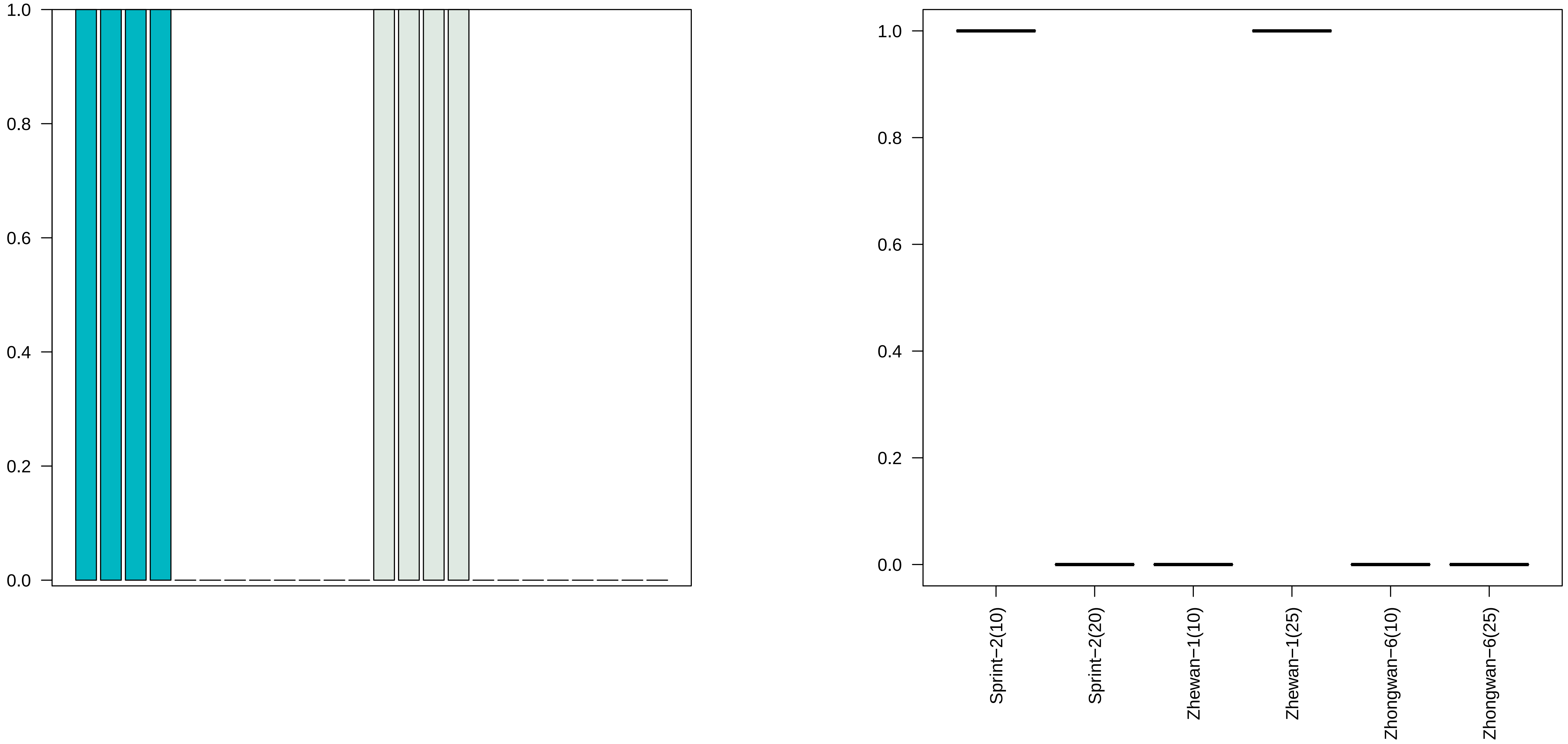

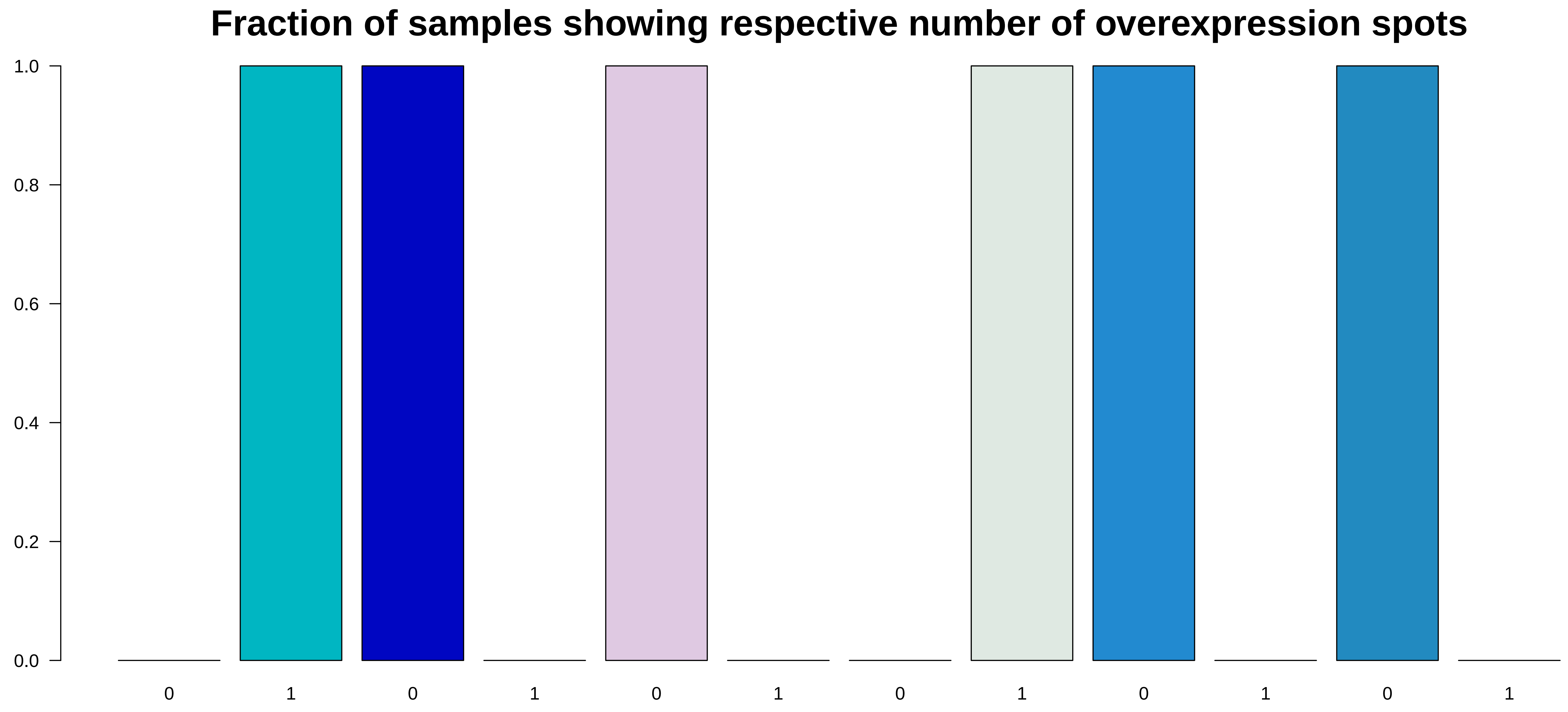

Fraction of samples showing respective number of overexpression spots
1.0
0.8
0.6
0.4
0.2
0.0
0
1
0
1
0
1
0
1
0
1
0
1

Fraction of overexpressed metagenes

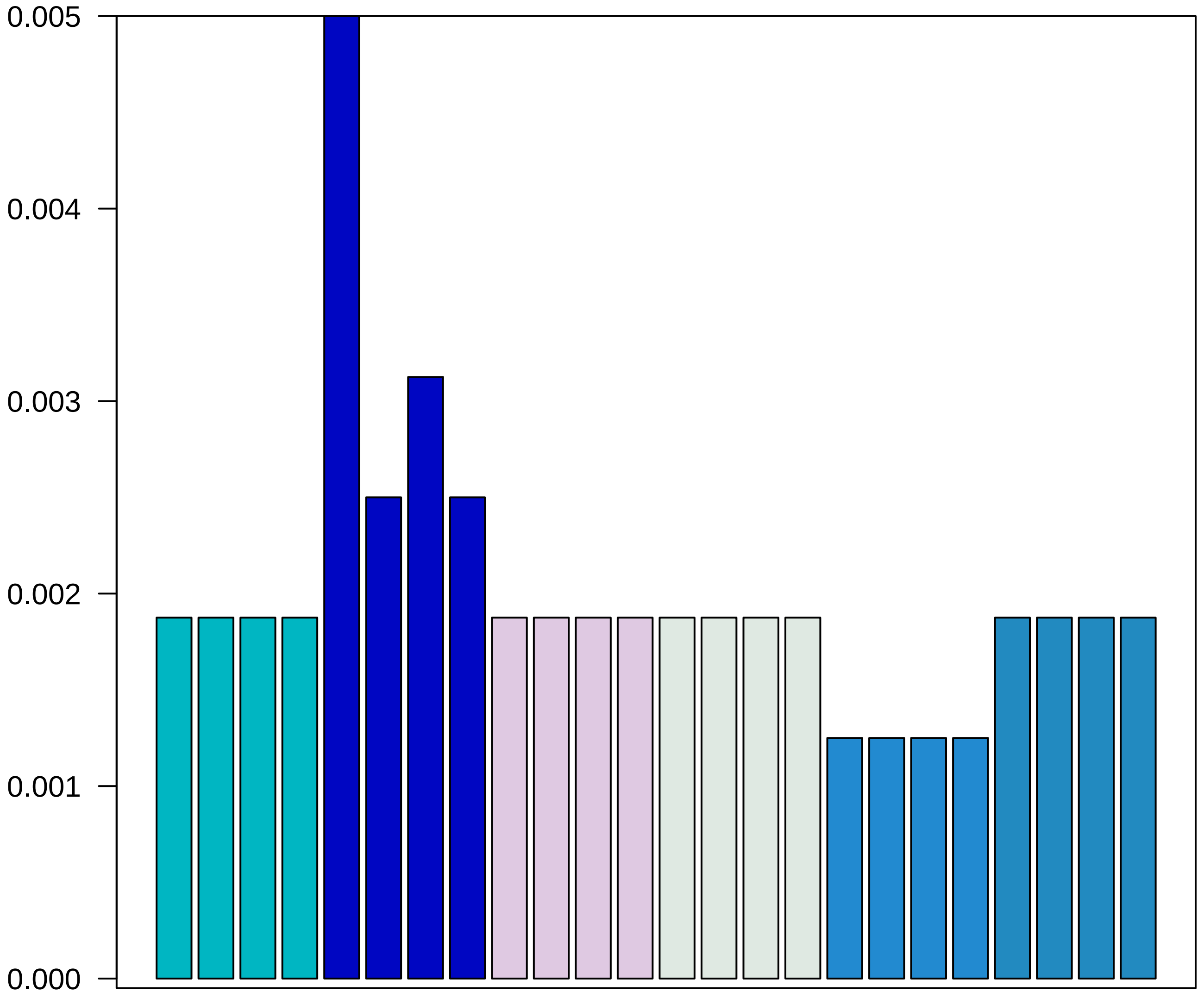

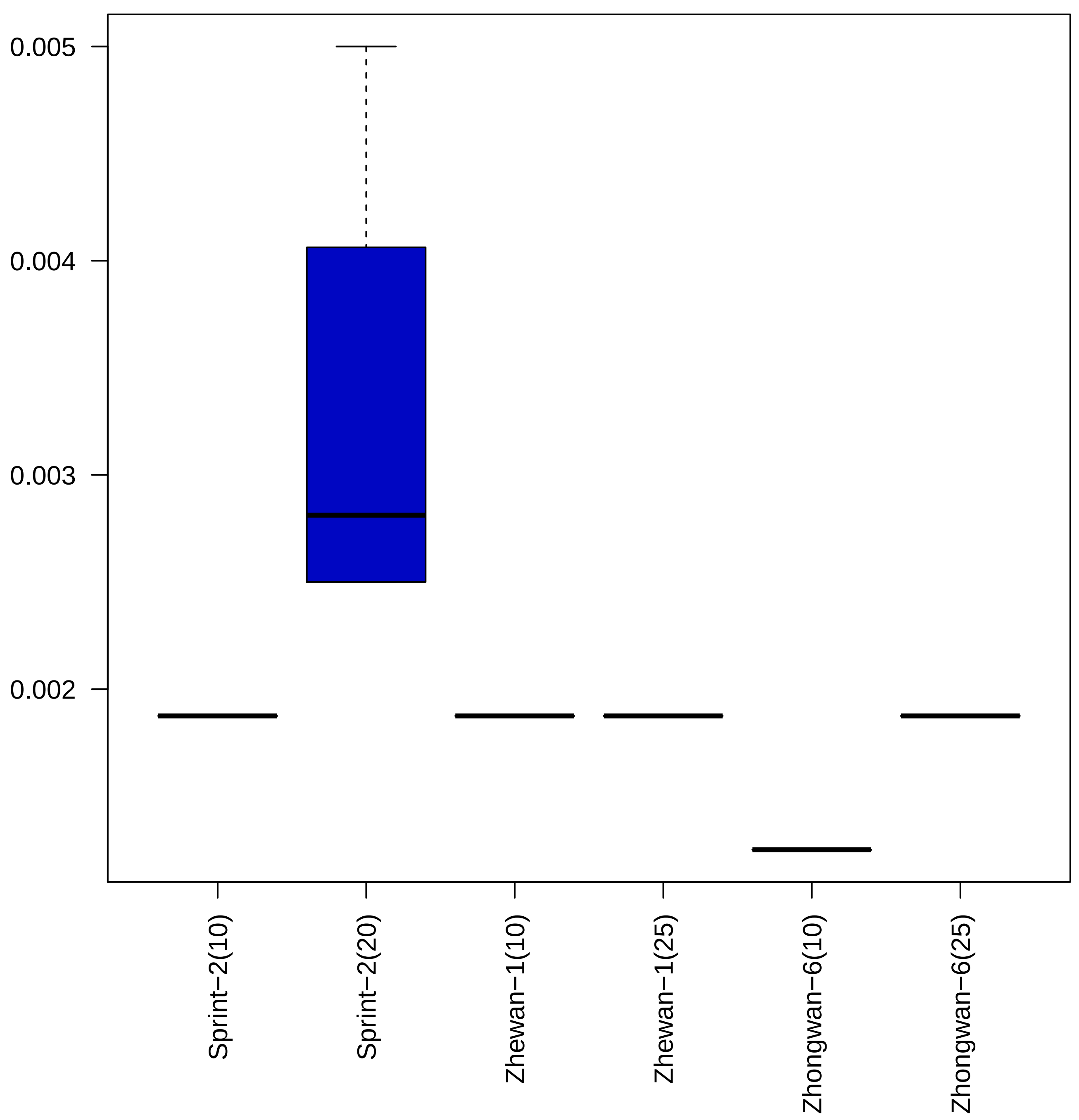

Length of borderline along overexpressed metagenes

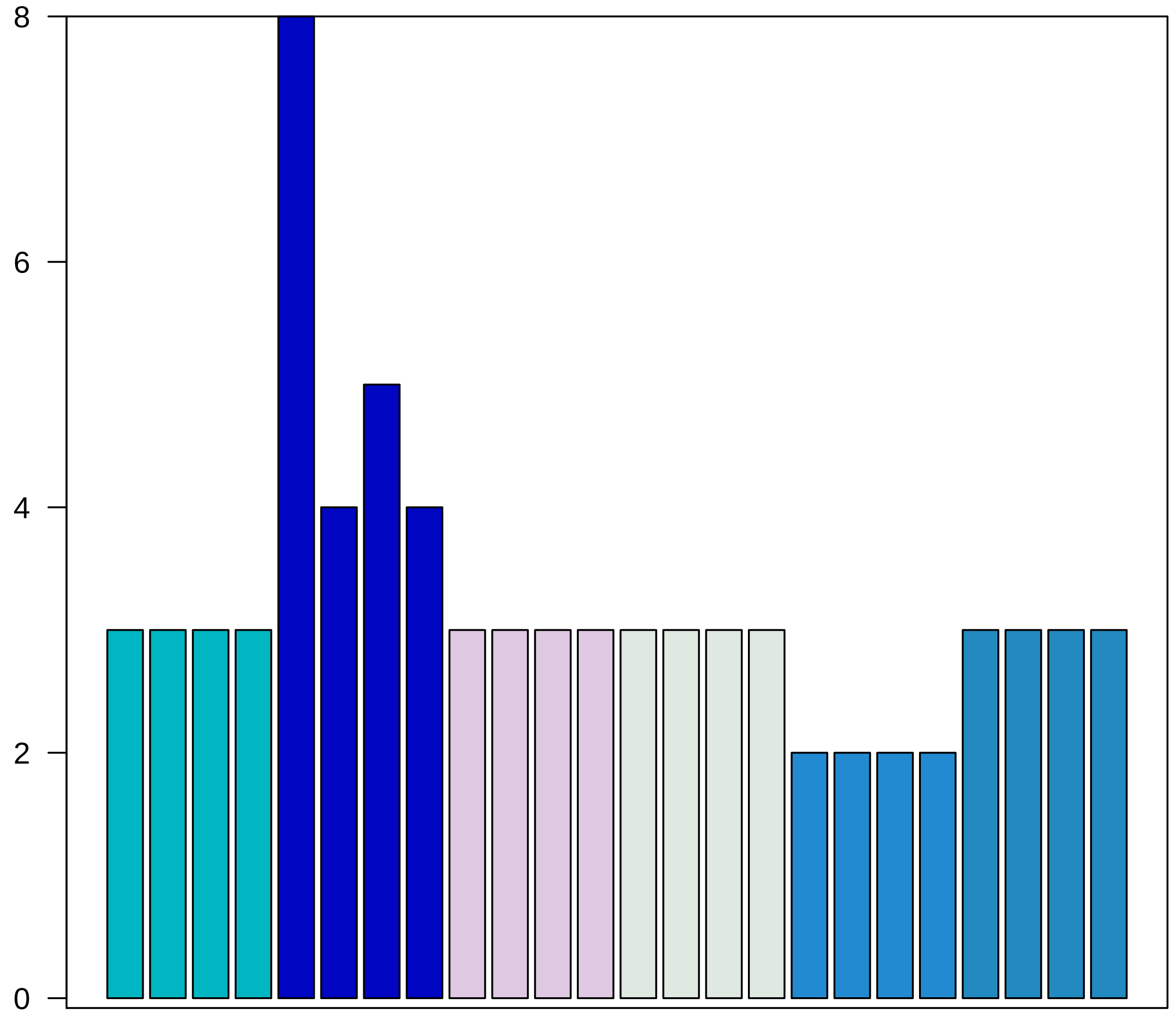

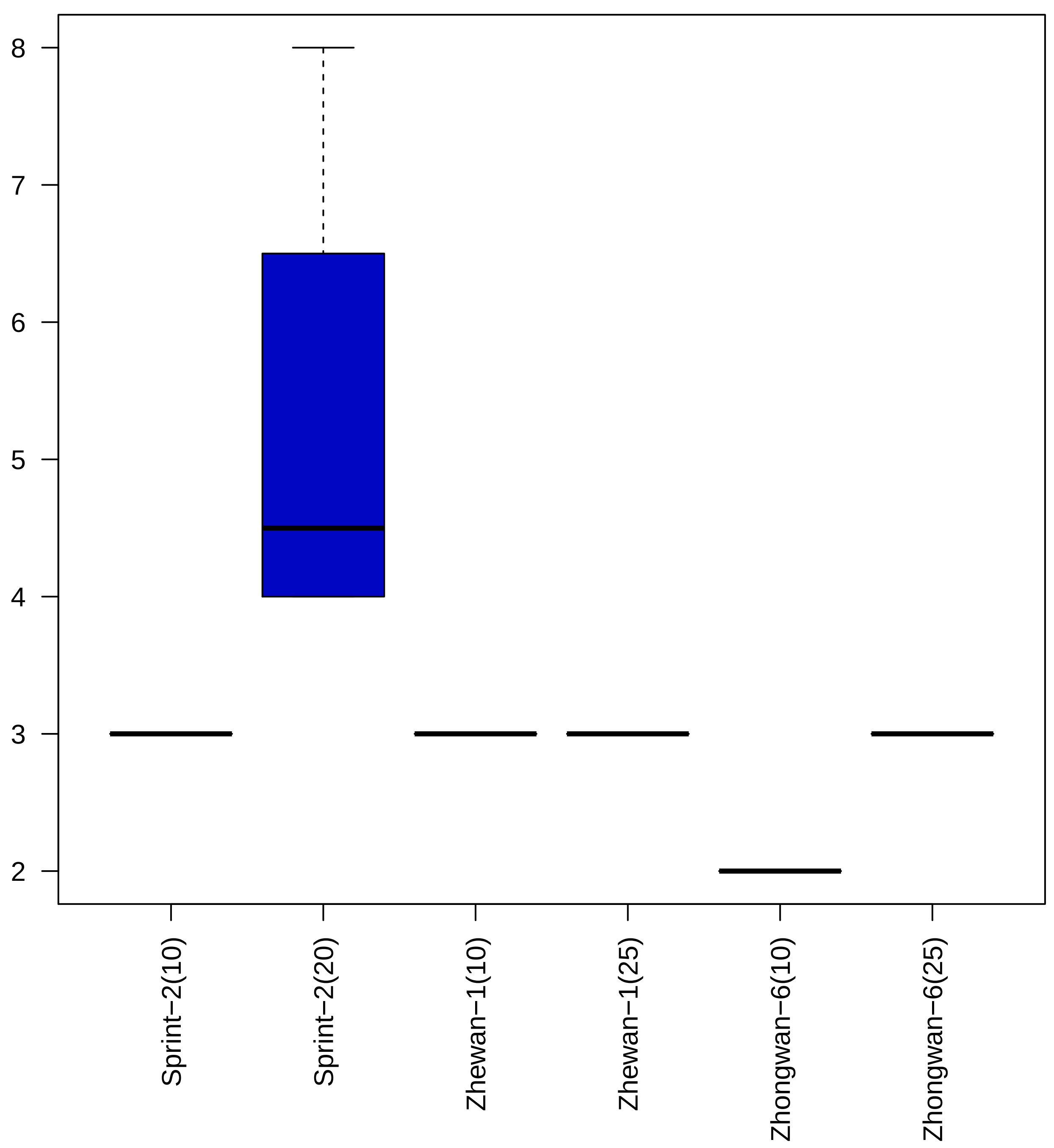

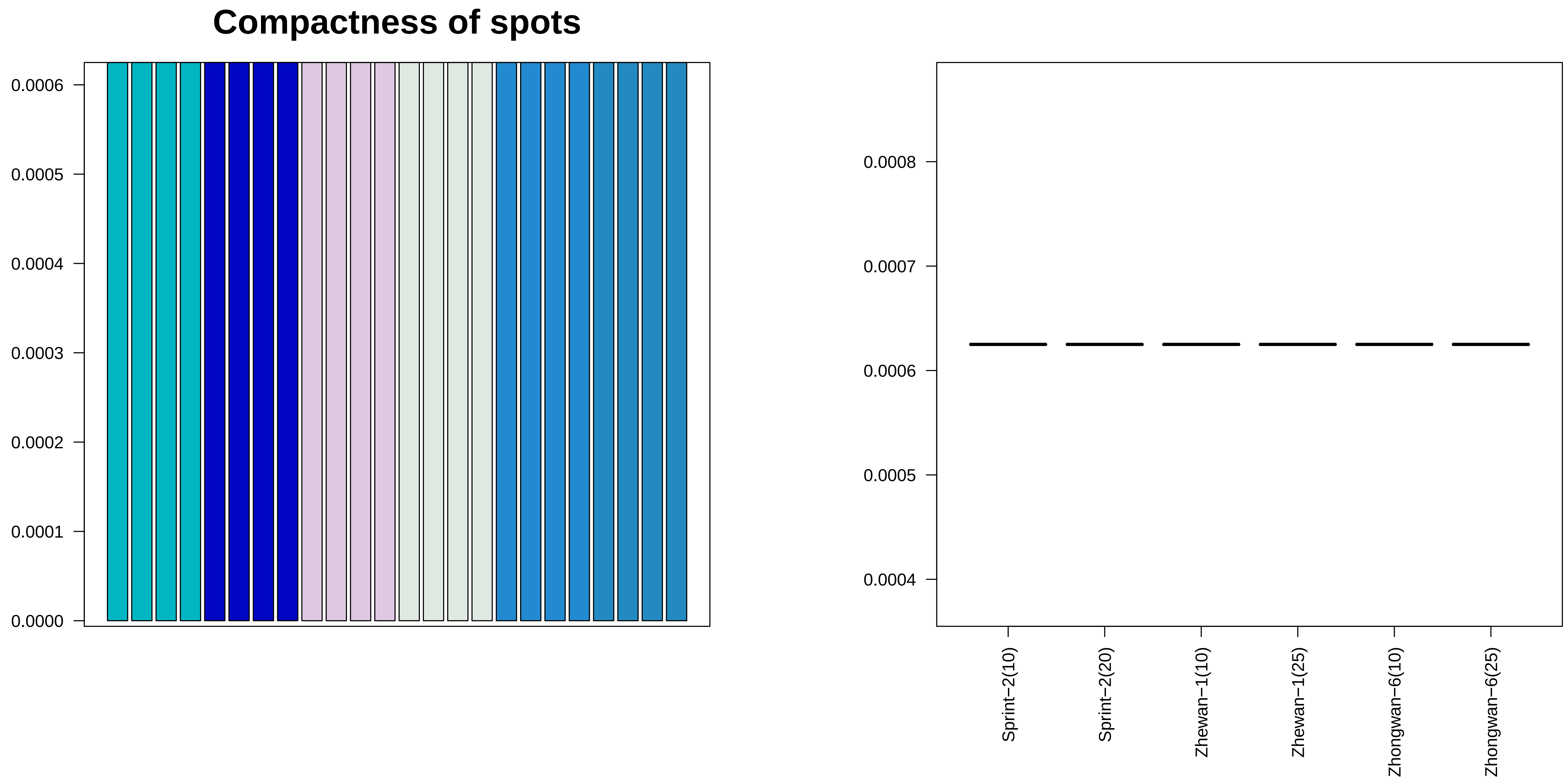

Compactness of spots
0.0006
0.0005
0.0004
0.0003
0.0002
0.0001
0.0000
0.0008
0.0007
0.0006
0.0005
0.0004
Sprint−2(10)
Sprint−2(20)
Zhewan−1(10)
Zhewan−1(25)
Zhongwan−6(10)
Zhongwan−6(25)

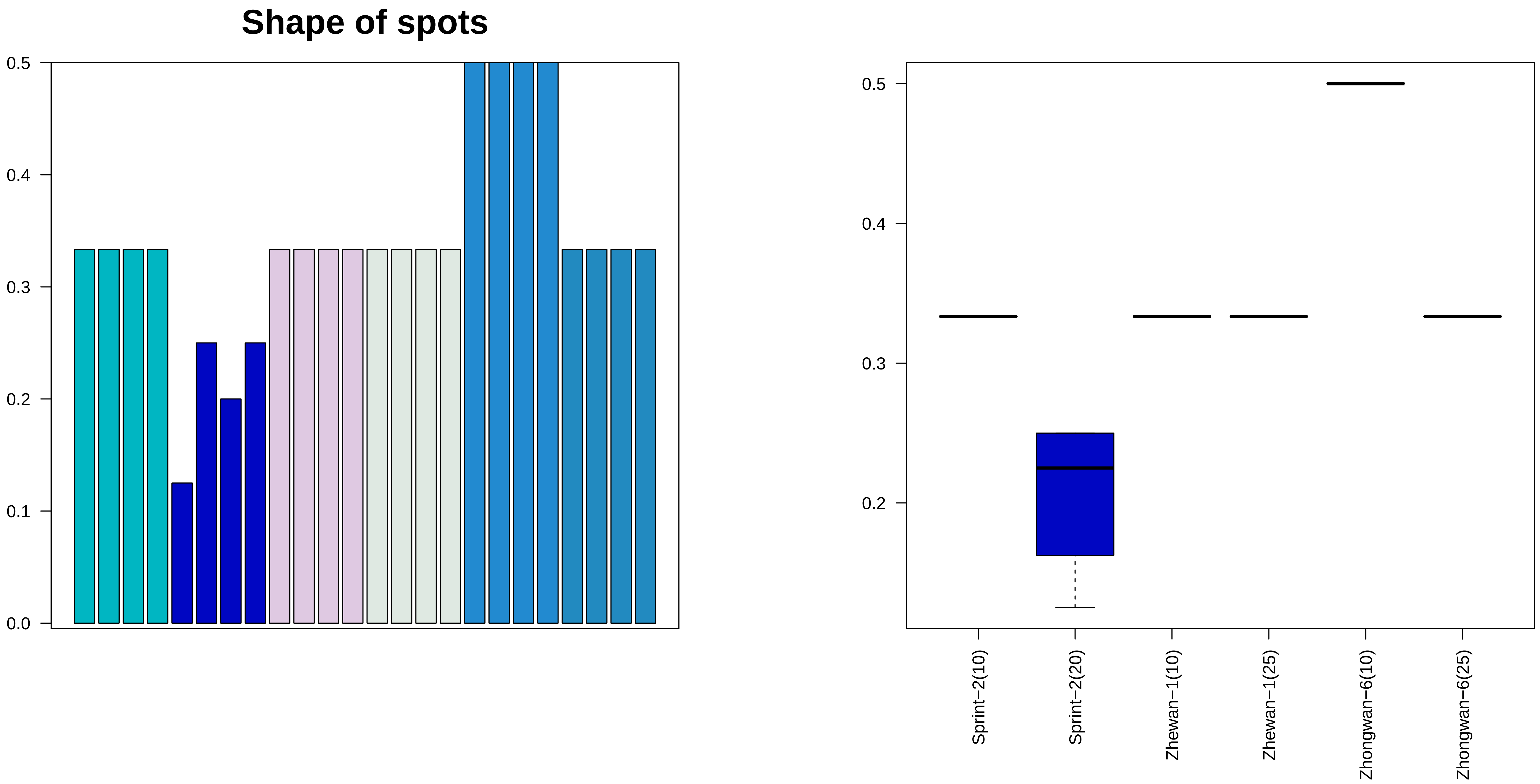

Shape of spots
0.5
0.4
0.3
0.2
0.1
0.0
Sprint−2(10)
Sprint−2(20)
Zhewan−1(10)
Zhewan−1(25)
Zhongwan−6(10)
Zhongwan−6(25)
